# Bio inspired general artificial muscle using hybrid of mixed electrolysis and fluids chemical reaction (HEFR)

**DOI:** 10.1038/s41598-022-07799-9

**Published:** 2022-03-07

**Authors:** Ramin Zakeri, Reza Zakeri

**Affiliations:** 1 Mechanical Engineering Department, Shahrood University of Technology, Shahrood, Iran; 2 Semnan Science and Technology Park, Shahrood, Iran

**Keywords:** Engineering, Mechanical engineering, Biochemistry, Biophysical chemistry

## Abstract

One of the issues in the field of soft-robotic systems is that how to create a fast displacement mechanism which it operates close to nature. This paper presents a deep study of hybrid of mixed electrolysis and fluids chemical reaction (HEFR) method for general applications, considering contraction/expansion of a single/multiple (taped) soft bio-inspired actuators in various conditions and a practical instance of a moving wing mechanism. This research extends the recent study of corresponding author’s team (Zakeri and Zakeri, Deformable airfoil using hybrid of mixed integration electrolysis and fluids chemical reaction (HEFR) artificial muscle technique. Sci Rep 11:5497, 2021) that previous study concentrated on just single bio actuator in deformable airfoil. This work offers a general artificial muscle which it employs the hybrid of mixed electrolysis (electrolysis module with 10 mL capacity without any separation of materials such as O_2_ or H_2_), two fluids for chemical reaction (sodium bicarbonate (NaHCO3 (s)) and acetic acid (CH3COOH (l))) and also multilayer soft skin bags (40 × 30 mm). The analyzed parameters are amount of displacement (contraction/expansion) over time (response time), the ratio of output force to total weight and extremely low expense of manufacturing. The main results are as follows: the released energy from 1 mL sodium bicarbonate, 10 mL acetic acid and a 12 V electrolysis module have ability to give a response time less than 1 s (25 mm expansion and 4 mm contraction) with 12 W power consumption and also bio actuator can easily displace a 250 g object (total weight of components is almost 33 g). Also, it has been shown that the response time of mixed electrolysis in the proposed inactive solution (without any fresh chemical reaction) will be nine times to pure water. In the active solution (refresh chemical reaction), response time of HEFR will be accelerated 2.44 times to pure chemical reaction. By applying the multilayer soft skin bags or soft actuators (multi contraction and multi expansion model), a practical movable flapping wing has been presented which a full cycle of flapping would take 2 s. The proposed method has ability to show a quick response time, without making any noise, very low construction cost and practical for general and frequent uses.

## Introduction

Nature has always been the source of inspiration in various sciences. In the field of artificial muscle, mimicking from natural contraction of a living muscle will be the best source for achieving the best design and manufacturing of an artificial muscle as well. By studying the physiology of a muscle, it can be seen that a displacement of a natural muscle is the series/parallel combinations of filaments/fibers. A filament is a combination of the two basic elements myosin and actin, when the myosin moves, it displace the actin which it will cause a contraction of muscle filament^1–3^. The required energy for such a displacement or contraction of the filament is gained by the breaking of a chemical bond that it originates from an electrical signal from a neuron motor. Such a complex mechanism ultimately creates a very fast and powerful movement that it will perform very well in complex environments, and we can see it in all living creatures^4–10^. There are numerous methods for production of displacement which most of them will have serious limitations, including high-power supply, very high weight compared to the output power, lack of flexibility at complex environments^11,12^.

Swamardika^13^ used servo motor for manufacturing a mobile robot with a robotic arm and wireless communication. They showed that moving in the different directions was possible but displacement on complex environment requires another approach in this regard. Also, hydraulic and pneumatic methods require at least a powerful and high weight pump or compressor which they will clearly make the noise pollution^14–16^.

Using Soft robotic, unlike hard robotics, it has taken a closer look at nature to solve this challenge, and many researchers have presented different methods^11^. One of the applicable methods in soft robotic is dielectric elastomer which by changing the electric energy to mechanical work, it has been able to provide a conspicuous displacement. Yang et al.^17^ used the dielectric elastomer as an artificial muscle to provide displacement for mimicking a cuttlefish movement. Although these methods such as dielectric elastomer or in general, electroactive polymers (EAP) have advantages in terms of low volume or weight but dependency to high voltage, low power output, far from natural mechanism, and low total efficiency are the main drawbacks of these methods^18^.

Researchers such as Lancia et al.^19^ suggested especial material which it is affected from lighting energy. Base on this material, lighting energy changes to mechanical energy and movements of molecular machines occur in micro-scale. This method, considering the low weight of the mechanism, this method has disadvantages such as output force is extremely low (mN) and the response time is so high. Schaffner et al.^20^ used the seamless fabrication of pneumatic silicone actuators which actuators can provide suitable displacement. Compressing air is an essential for inflation which dependency to high power compressor is a drawback of this type of methods. Miriyev et al.^21^ applied the self-contained electrically driven soft actuators which they used shape memory alloys. This method also needs high voltage and contraction over time is so slow. Recently, Keplinger^22–24^ applied new method, called HASEL, by combination of electrostatic actuators and hydraulic effect of fluid, the conspicuous displacement has been reported in short period of time while the need for high voltage is an essential for this technique.

Considering the natural mechanisms, the use of chemical materials and stimulation of electrical signal are an integral part of such mechanisms^1,2^. Martinez^25^, for the first time, applied chemical reaction of glucose and oxygen to produce energy and these materials could move an artificial muscle. It has been shown that the chemical compound has enough potential for generating electricity like a battery, which generated electricity can be used for moving an electro active polymer muscle. Except the restrictions of EAP was mentioned earlier, based on the nature, chemical energy should be directly changed potential energy of materials into mechanical energy and development of the presented mechanism should be performed. The Fluid-driven origami-inspired artificial muscles is a proper mechanism which fluid pressure is directly changed into mechanical work by air bags, however dependency to high pressure compressor is still a disadvantages of this method^26^.

Also, the materials-based actuators by the charge injection have high performance, for example, Wu^27^ applied the phosphorene electromechanical actuators which this method enjoys the high volumetric work density (three orders of magnitude larger than natural muscle). Wu^28^ also used Ti_2_C MXene which this material can be applied for high-performance nanoelectromechanical actuators. High-Performance Graphene Oxide Electromechanical Actuators is another material—based method which it can provides high strain^29^. These methods are so interesting method but they are applicable more in nano/micro scale and more investigation for practical cases would be necessary in industrial macro scale.

Inspired by nature as a complete reference, Zakeri^30^ in previous study, introduced HEFR (Hybrid of Mixed Electrolysis and Fluids Chemical Reaction) technique for the first time to provide a deformable airfoil. In this study, some main criteria for presenting a general artificial muscle (HEFR) with considerable displacement and short response time have been considered including effect of different electrolytes, response time, ratio of output force to total weight, lightness and mimicking the part of mechanism from nature to lead a new study for single soft actuator, multiple soft actuators (taped actuators) and this approach apply for a moving wing mechanism.

## Results and discussion

According to the introduction, one of the drawbacks of the existing methods is that the available mechanisms are not close to the natural muscle, thus some aspects of mentioned methods such as displacement in proper response time under loading condition, ratio of output force to total weight have lower efficiency compared to natural muscles. In this section, the mechanism of a natural muscle presents first explicitly and then the proposed artificial muscle mechanism is expressed for practical uses. In the next sections, we will test the proposed artificial muscle or HEFR and examine single soft actuator in different modes. Then, the results will be developed into multiple (taped) soft actuators or multiple artificial myosins which they can be developed for general applications and finally the movement of a flapping wings will be investigated based on the obtained results from previous sections as a practical application of proposed method.

### Principle of natural muscle contraction and proposed HEFR model

The contraction of a muscle is done by thin strands, called fibers, and each fiber is made up of smaller fibers or filaments. One of the most respected theories of biology in the field of filament contraction performance is sliding filament theory. According to this valid theory, muscle contraction is attributed to two members including myosin and actin. The wave motion of the myosin will cause sliding movement of actin. The mechanism of this contractile action is that first the signal from the motor neuron (called action potential) will release calcium ion from the sarcoplasmic reticulum which not only does it provide the binding sites on actin for myosin but also it stimulates myosin by changing ATP (Adenosine tri-phosphate) to ADP (Adenosine diphosphate) and as a result, myosin will move and bind to actin (called cross bridge) and motion of myosin can pull the actin for contraction purpose. In order to relax the muscle, calcium return to the sarcoplasmic reticulum, then myosin moves to its original position (broken the cross bridge), and actin will lose its contractile state and strength.

**Figure 1:**
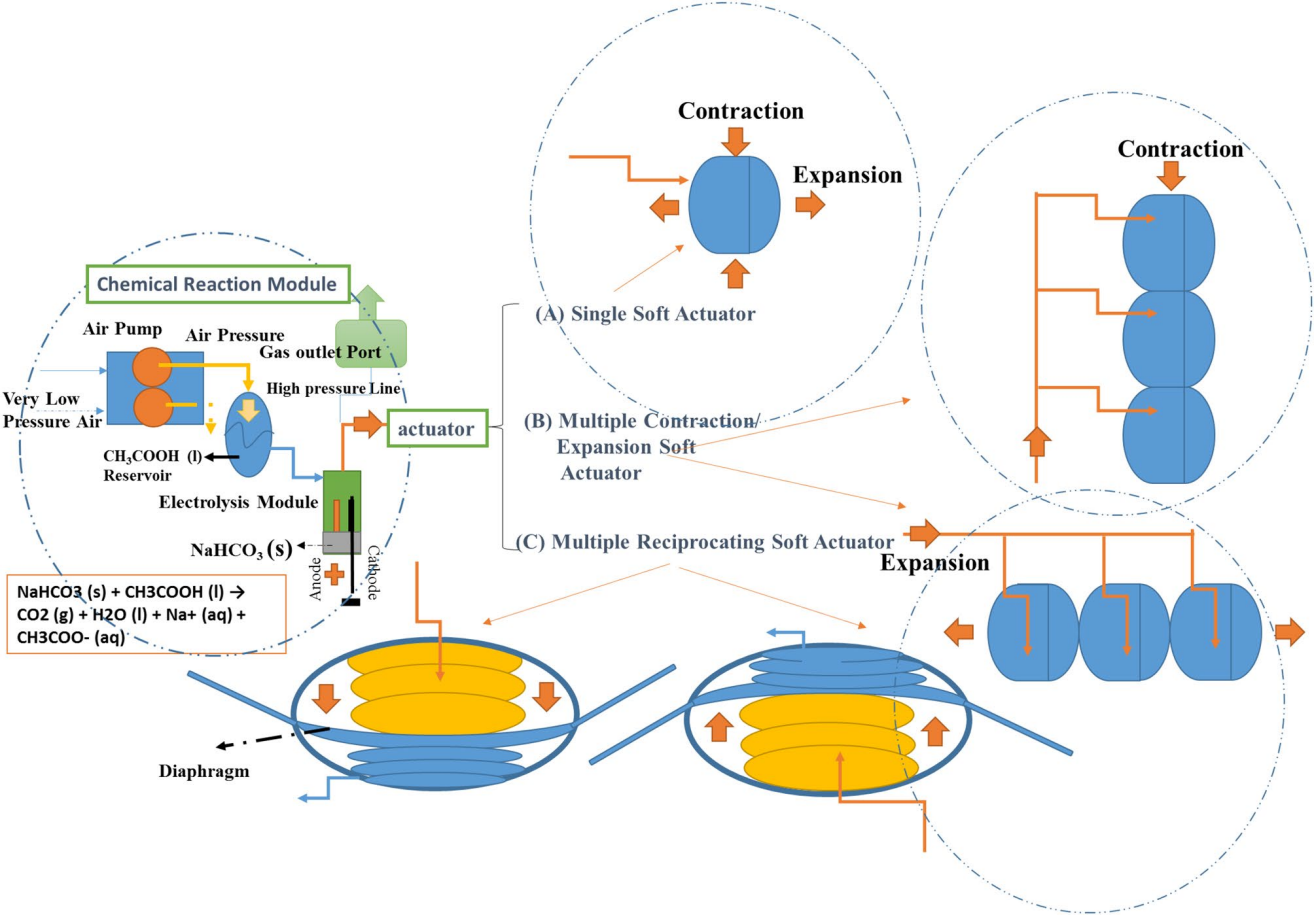
Proposed model for HEFR (hybrid of mixed electrolysis and fluids chemical reaction) artificial muscle. Single soft actuator **(A)** for achieving a single contraction/expansion, multiple soft actuators** (B)** for having higher contraction/expansion and multiple reciprocating soft actuators **(C)** for forming reciprocating motion.

The presented method in this paper is based on part of the natural process. Natural contraction of myosin will be achieved by releasing calcium ions and stimulating myosin to convert chemical energy to mechanical energy and displacement will be formed. According to Fig. 1, the energy source for the movement of the soft actuator is due to hybrid of chemical reaction and the electrolysis of the fluids. It is shown that the combination of these two effects provide a very powerful and desirable movement. Then, the released energy is directed to the high pressure fluid collection bag or soft actuator to create contraction by the swollen operation. In this paper, three test cases including single fluid collection bag (soft actuator), multiple fluid collection bags (soft actuators) and a moving wings mechanism are considered respectively.

**Figure 2:**
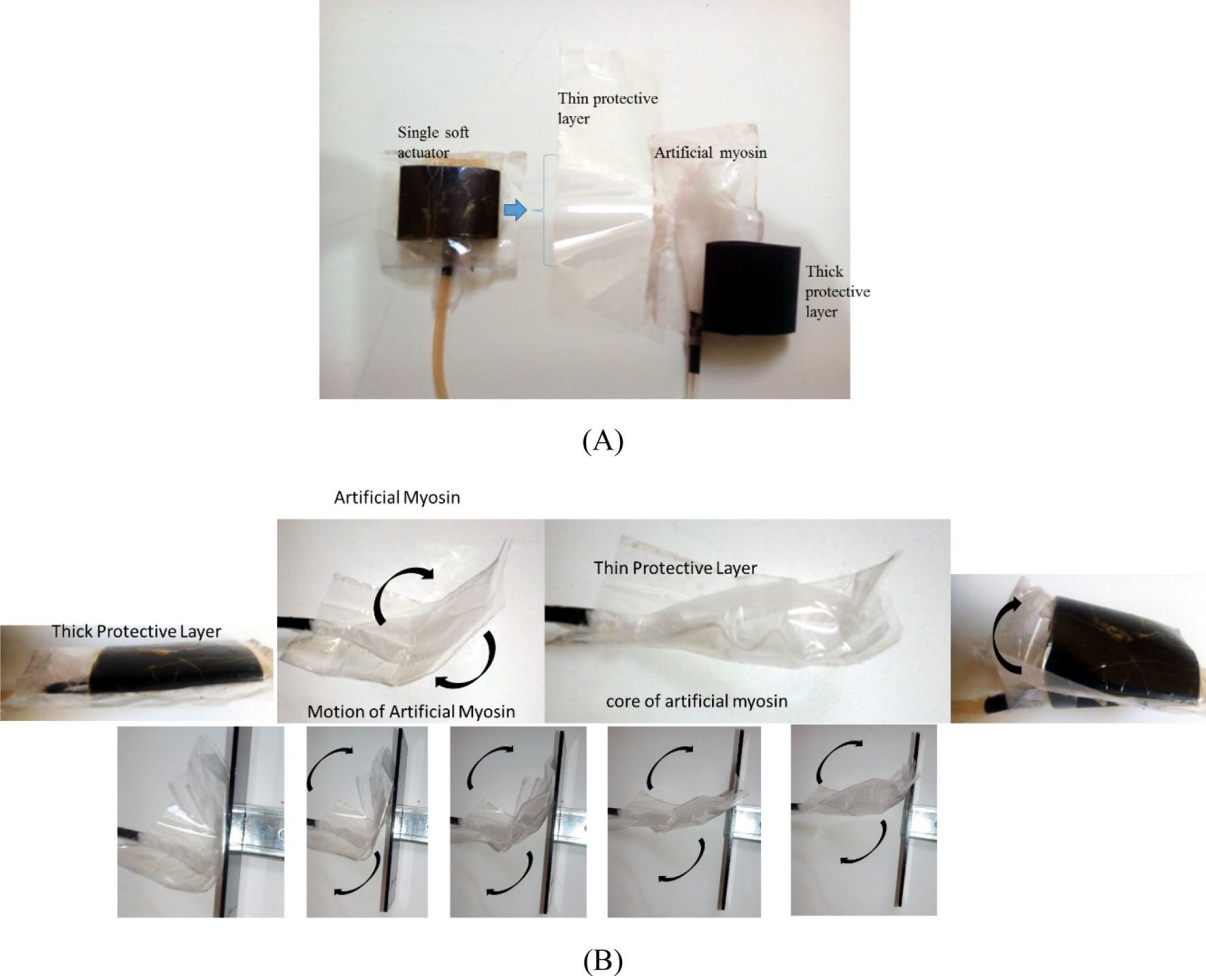
Soft actuator construction and inflation of artificial muscle. Different layers of soft actuator including thin and thick layers **(A)** and changing the wave motion of artificial myosin to expansion/contraction **(B)**.

As can be seen in Fig. 2, inside of soft actuator or fluid collection bag, which is inspired by motion of natural myosin, the thin artificial myosin moves in form of wave motion and its motion will be changed to swelling action by two protective soft and thick rubber layers. Contraction of soft actuator from one side and expansion from another side provide a significant displacement which response time and produced power depend on entry energy into soft actuator and also total displacement can be multiple using multi-soft actuators. Thus, soft actuator consists of several layers with different flexibility to safely collect fluid under pressure, respectively, the inner layer is very thin and impermeable, and the middle layer is thin but with high strength and the outer layer is very strong and thick. The bonding several coating layers can increase their resistance against any leakage because fluid pressure in actuator is enough high. In order to release the muscle, the compressed fluid is discharged from bags or soft actuators and they return to their original position and the artificial filament is released, which it is equivalent to the release of the natural filament.

**Figure 3:**
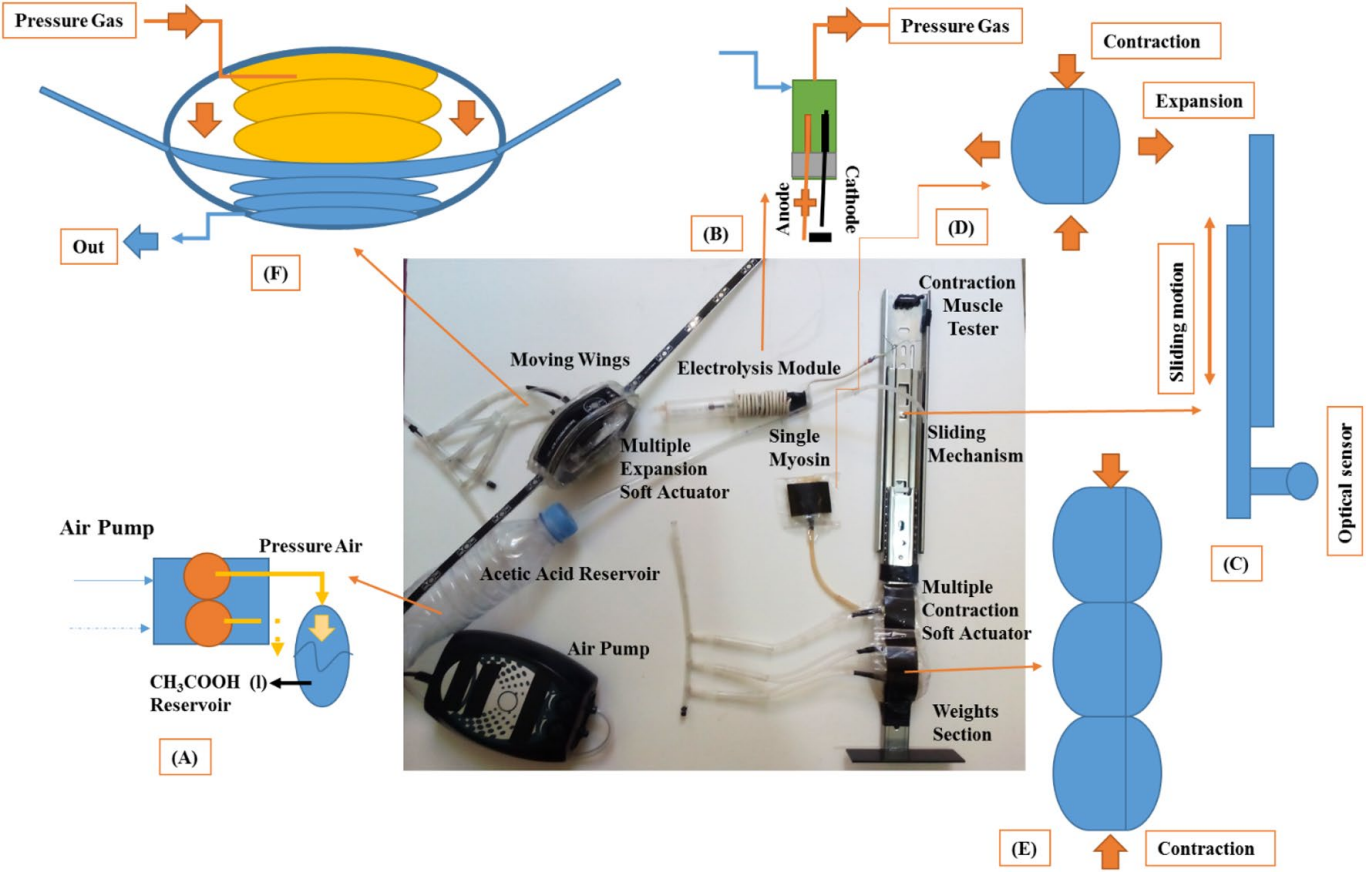
Experimental set up of HEFR (hybrid of mixed electrolysis and fluids chemical reaction) including air pump for pressurization of the acetic acid reservoir **(A)**, mixed electrolysis module/chemical reaction tank **(B)** for swelling of soft actuator, contraction muscle tester for testing of soft actuator behavior **(C)**, single soft actuator **(D)**, multiple soft actuators **(E)** and moving wing which convert pressurized fluid into mechanical motion **(F)**.

Figure 3 shows the experimental equipment for handling this research and dependency between different parts was illustrated in Fig. 1. The potential energy in materials are changed to chemical energy by two mechanisms of electrolysis and chemical reaction. The chemical reaction is a combination of sodium bicarbonate (NaHCO3 (s)) and acetic acid (CH3COOH (l)) which according to the chemical equation of NaHCO3 (s) + CH3COOH (l) → CO2 (g) + H2O (l) + Na + (aq) + CH3COO- (aq), will cause the release of a large amount of carbon dioxide gas^31^. The electrolysis mechanism also consists of two simple metal (iron) electrodes. The reason for the simplicity of these two electrodes would be because of the low cost and the possibility of easily replacing them. Also, the air pump is used to pressurize the acetic acid reservoir and discharge into electrolysis module. In the floor of the electrolysis module, it is possible to inject the sodium bicarbonate which it will cause the mentioned chemical reaction. High pressure produced gases (combination of gases from ion and cathode electrodes, called mixed electrolysis) enter the actuation section. Three types of actuators are considered for HEFR mechanism including (Fig. 3):

- A fluid collection bag with different layers which it performs as a soft actuator to study the behavior of response time in different states including pure electrolysis for different solutions, pure chemical reaction and hybrid of electrolysis and chemical reaction.
- Taped soft actuators or multiples contraction artificial muscles (fluid collection bags) has been employed for providing large displacement (contraction or expansion) which the performance will be investigated by contraction muscle tester which it is explained in next section.
- Reciprocating movable wings designed and built from a combination of two sets of taped soft actuators which they are opposite each other for providing reciprocating motion.

### Volume changes of single soft actuator

**Figure 4:**
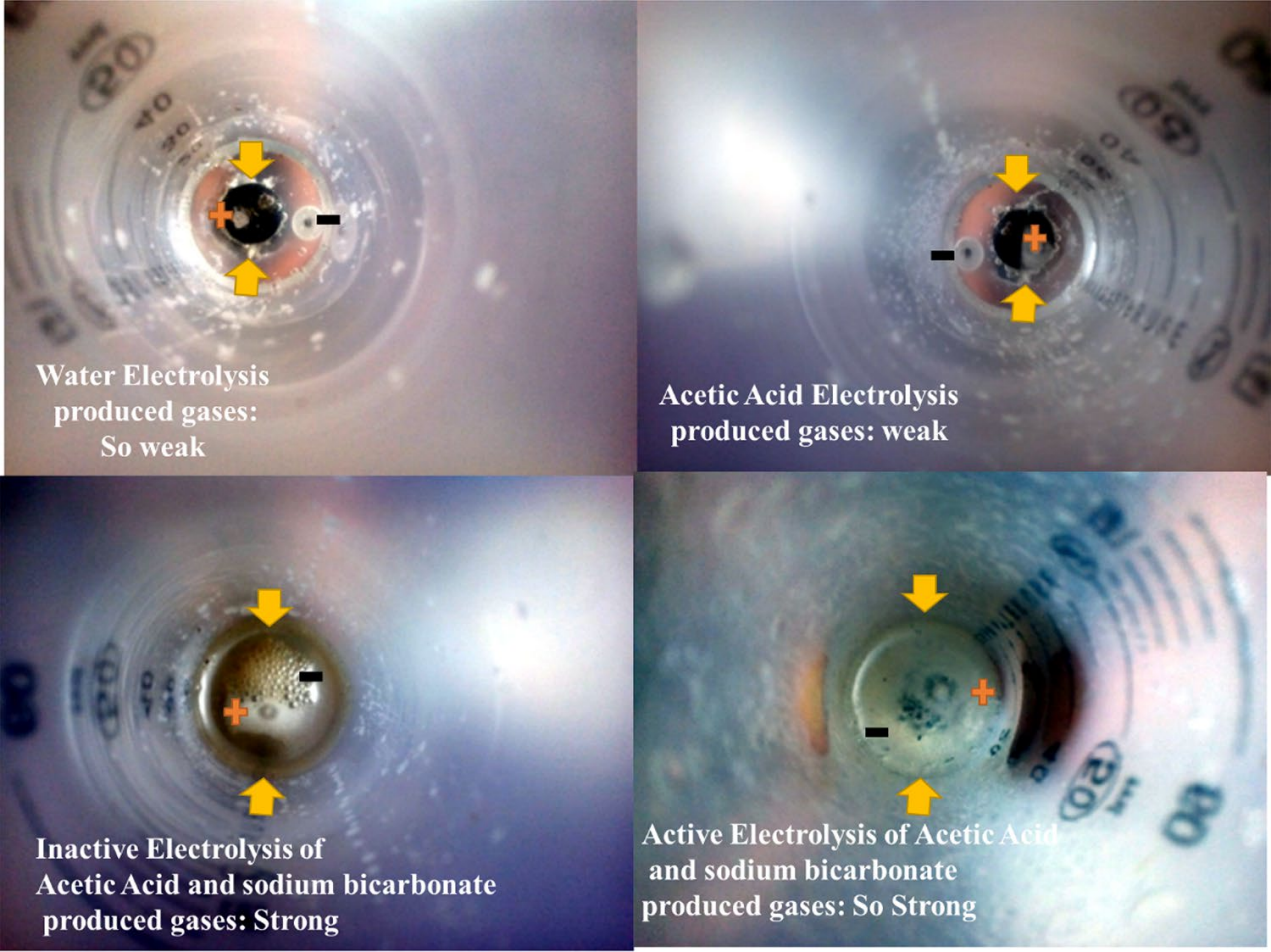
Comparison of electrolysis of different electrolyte solution (water, acetic acid, inactive mode and active mode of acetic acid and sodium bicarbonate) and volume of produced gases.

Before emphasizing on the application of this method, the performance of soft actuator in different cases should be investigated for finding the response time, power consumption and output force to weight ratio. Our aim is that the swelling rate of fluid collection bag or bags (soft actuator) should be determined for different modes. The modes include pure electrolysis, pure chemical reaction and combination of them. From electrolysis aspect in Fig. 4, different volumes of discharged gases from decomposition of molecular bonds can be clearly detected by comparison of four different electrolyte solutions in electrolysis process including pure water, pure acetic acid, inactive acetic acid and sodium bicarbonate and active mode of them. The volume of discharged gases can surly influence on response time, therefore, the effect of three modes are studied including pure electrolysis (for different solutions including water and acetic acid), pure the chemical reaction (acetic acid and sodium bicarbonate) and the hybrid model of mixed electrolysis with inactive/active chemical reactions. Please note that our purpose from inactive/active chemical reaction is that in inactive state, mixing a little amount of sodium bicarbonate (1 mL) with acetic acid are carried out and the solution should have sufficient rest for several minutes but in active chemical reactions mode, a little amount of sodium bicarbonate with acetic acid are mixed and immediately solution should be used for achieving proper displacement in short time. The behaviors of these states are presented in the following sections, respectively.

#### Swelling of soft actuator by pure electrolysis

**Figure 5:**
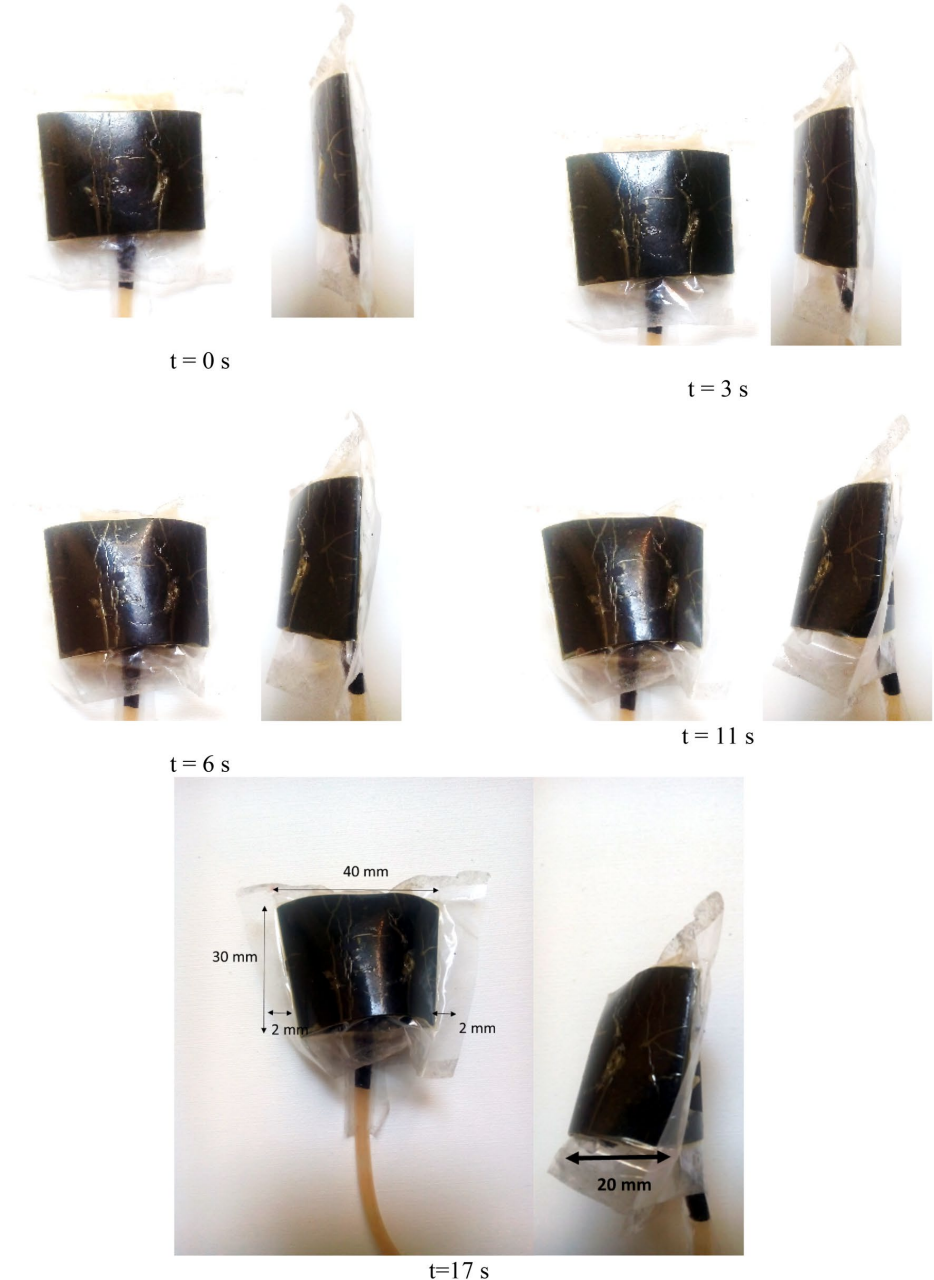
Volume changes of artificial muscle over time using pure electrolysis of inactive solution of sodium bicarbonate with acetic acid (inactive mode) in constant voltage 12 V.

In this section, we examine the effect of pure mixed electrolysis (without any separation between oxygen and hydrogen) of several substances that they would produce gas compounds in electrolysis process and it will eventually cause swelling of soft actuator (air bag). Due to inflation of soft actuator eventually leads to displacement over time for the inactive mode of sodium bicarbonate with acetic acid as shown in Fig. 5. Also, in mentioned figure, total displacement for a soft actuator from contraction aspect is 4 mm and expansion (swelling) is 20 mm. Response time, power consumption and output force to total weight ratio completely depend on voltage and electrolyte solution. The optical method as mentioned in the “Principle of natural muscle contraction and proposed HEFR model” was used with accuracy of 1 mm for swelling measurement.

**Figure 6:**
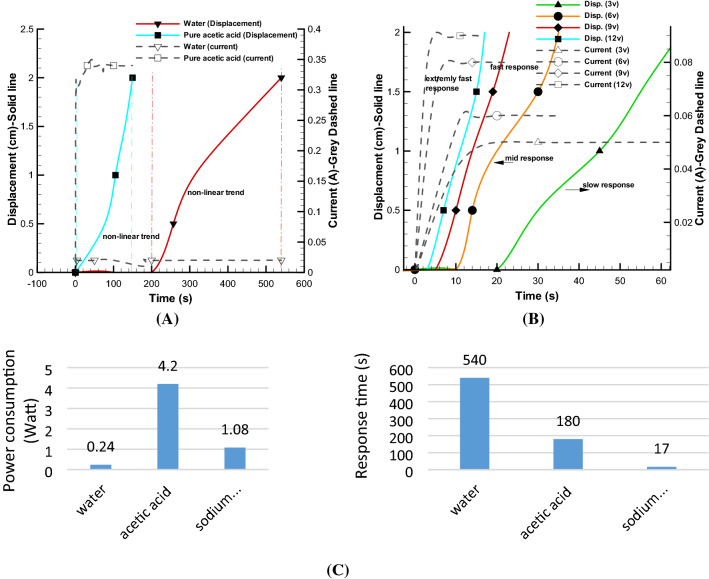
Pure electrolysis of different electrolyte solutions and expansion of soft actuator volume over time. Comparison of displacement-response time and current consumption of pure water, pure acetic acid at 12 V **(A)**, inactive solution of sodium bicarbonate and acetic acid (inactive mode) at 12, 9, 6, 3 V **(B)** and bar chart of power consumption and response time of single actuator for different electrolytes **(C)**.

Initially, water electrolysis was used to create displacement in a soft actuator. The geometrical characteristics of the electrolysis module and the soft actuator are given in Table 1. Figure 6 (A) illustrates the behavioral trend of displacement–time changes for pure water and artificial muscle displacement has been recorded for 12 V and current consumption of 0.02 A. The volume changes in pure electrolysis mode is very slow and non-functional. After about 9 min, a cell (soft actuator) moved just 20 mm. In general for water test case, although by increasing of voltage, the current consumption will raise, but such the results cannot be applied to a functional artificial muscle due to long electrolysis process (weak displacement). Thus, response time is so slow (9 min), power consumption is so low (0.24 Watt) and this experiment cannot be a practical sample for artificial muscle test case obviously.

**Table 1:** Characteristics of electrolysis module and the soft actuator.

Characteristics	Mixed electrolysis	Characteristics	Actuator
Size (cylinder)	18 (mm) $\times$ 65 (mm)	Size (single)	30 $\times$ 40 $\times$ 4 (mm)
Electrode size (cylinder)	15 mm $\times$ 1.5 (mm)	Size (multiple contraction)	30 $\times$ 120 $\times$ 4 (mm)
Voltage	3–12 (V)	Size (multiple expansion)	30 $\times$ 40 $\times$ 12 (mm)
Current	0.01–1 (A)	Displacement (single)	20 (expansion) $\times$ 4 (contraction) (mm)
Ratio of sodium bicarbonate to acetic acid	1/10	Displacement (multiple contraction)	20 (expansion) $\times$ 12 (contraction) (mm)
Distance between electrodes	1 (mm)	Displacement (multiple expansion)	60 (expansion) $\times$ 4 (contraction) (mm)

The reason for this slow decomposition process is that the strong bond between hydrogen and oxygen is not suitable for such decomposition with this low relative voltage. There is a non-linear trend which the total rate of produced mixed gases are increased due to raising the temperature of water but the process is still so slow. Also, although by increasing the voltage and consequently current will cause the response time becoming shorter but the work efficiency decreases significantly. To further investigate the effect of materials for decomposition in Fig. 6A, the acetic acid is applied since the bond between hydrogen and oxygen in this material is weaker, the decomposition is also faster and the volume of released gases will be higher. Conversely, the volume of the cell will change faster and non-linear trend is achieved faster rather than pure water. According to the same diagram, the same displacement of 20 mm has been obtained in about 3 min considering higher current value (0.35 A) but compared to nature, the results are still undesirable because the swelling process is still so slow. Thus, as results of the response time (3 min) and power consumption (4.2 W), electrolysis improvement for artificial muscle application is vital.

In the previous two cases, the test was not performed in lower voltage because the results were not proper for artificial muscle. The materials with weaker molecules bond will accelerate the reaction and consequently stronger electrolysis. Adding the little amount of sodium bicarbonate substance with acetic acid, considering this solution should be put to rest for a few minutes, consequently electrolysis process will have a very significant results. Using the mentioned solution at voltage of 12 V and 0.09 A (1.08 W), a displacement of about 2 cm is obtained in less than 17 s in mentioned Fig. 5. In other words, the response time not only improve to 90% compared to previous test case but also, the power consumption conspicuously decreases around 74%.

Also, for the inactive mode of sodium bicarbonate with acetic acid in lower voltage (3, 6 and 9 V), the experiments were repeated as shown in Fig. 6B. At lower voltages, the time has increased to some extent, and at the lowest voltages, 3 V and current consumption of 0.05 about 60 s are needed to provide proper displacement. From the results of this article and Fig. 6C, it can be reported that the use of the proposed solution will improve the electrolysis process for artificial muscle applications.

#### Swelling of soft actuator by mixed electrolysis and active chemical reaction

**Figure 7:**
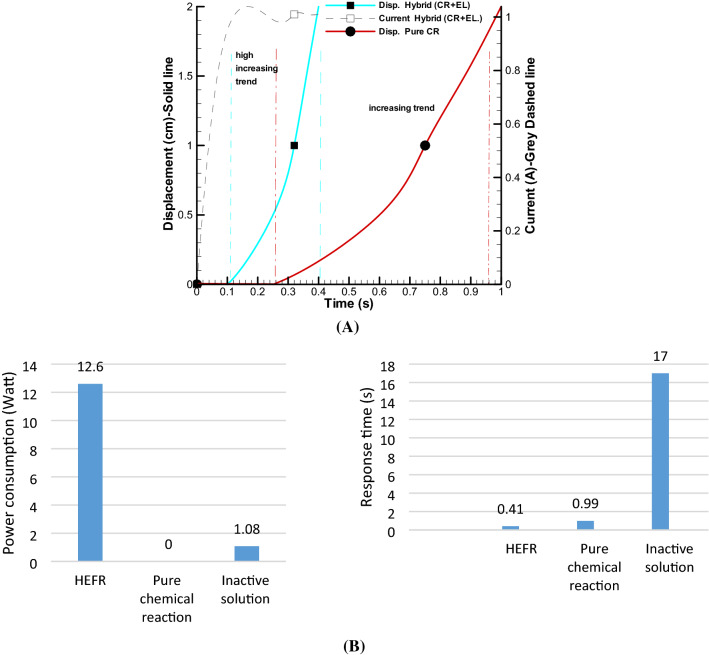
Displacement of soft actuator over time by pure chemical reaction (CR) and hybrid of chemical reaction and electrolysis (CR + EL, active mode) in constant voltage 12 V **(A)** and bar chart of power consumption and response time of single actuator for different modes **(B)**.

From the previous section, the inactive mode of mentioned solution has faster response than electrolysis of pure substance. It is clear that produced gas can affect on contraction/expansion of artificial muscle, thus, the effect of pure chemical reaction, hybrid model of electrolysis and fluid chemical reaction (HEFR) should be compared together. At the first, pure chemical reaction is studied in two statuses:

In this case, two substances including acetic acid and sodium bicarbonate are combined and the outlet valve is opened and after one minute the valve is closed and we will not have any result or displacement. In other words, the volume of produced gases after one minute are very small and cannot change volume of bags. It should be noted that the effect of electrolysis has not been used in this experiment. In the next experiment, the gas outlet valve is completely closed and with the entry of the acetic acid and sodium bicarbonate in less than a second, a strong reaction is performed and a displacement of 20 mm is created as shown in Fig. 7A. It should be noted that such an unsteady reaction occurred immediately and stopped after a short time, and re-production of this reaction would reduce the final efficiency because we have to consume a lot of chemical materials.

Combination of electrolysis and chemical reaction can solve mentioned problem from two aspects. Due to mixing the materials, the bond between atoms become so weak and it has desirable potential for electrolysis process. Beside the short response time, the power consumption of electrolysis process is lower that acetic acid. It is easy to conclude that the combination of the two methods will have a very significant result, considering the entry of small amounts of chemicals, and electrolysis of materials is a powerful and continuous process that it does not need any fresh chemical reaction. Also it enjoys shorter response time compared to a slow pure materials.

From another aspect, extremely quick response time will be possible by hybrid effect of active chemical reaction and electrolysis as an unsteady and fast mode. Figure 7A shows a rapid change, less than 0.5 s in 12 V and around 1 A current consumption, which highly nonlinear trend occurs in initial time of reaction. It is clear that this method presents a proper method for rapid and strong displacement of artificial muscle. Also, in Fig. 7B, bar chart of comparison of pure chemical reaction, inactive mode of electrolysis, and HEFR are depicted which fast response time of less than 0.5 s and power consumption of almost 12 W for a single soft actuator are reported by HEFR method.

Based on the obtained results, it can be concluded that for rapid and powerful instantaneous contraction or expansion, the active hybrid method (with consuming the chemical materials) is a proper choice and the use of the inactive hybrid mode (without consuming the chemical materials) can be used to achieve contraction or expansion with a lower rate. In order to increase the displacement, it is necessary to present a new and practical method, which will be explained in the next section.

### Contraction of multiple soft actuators

**Figure 8:**
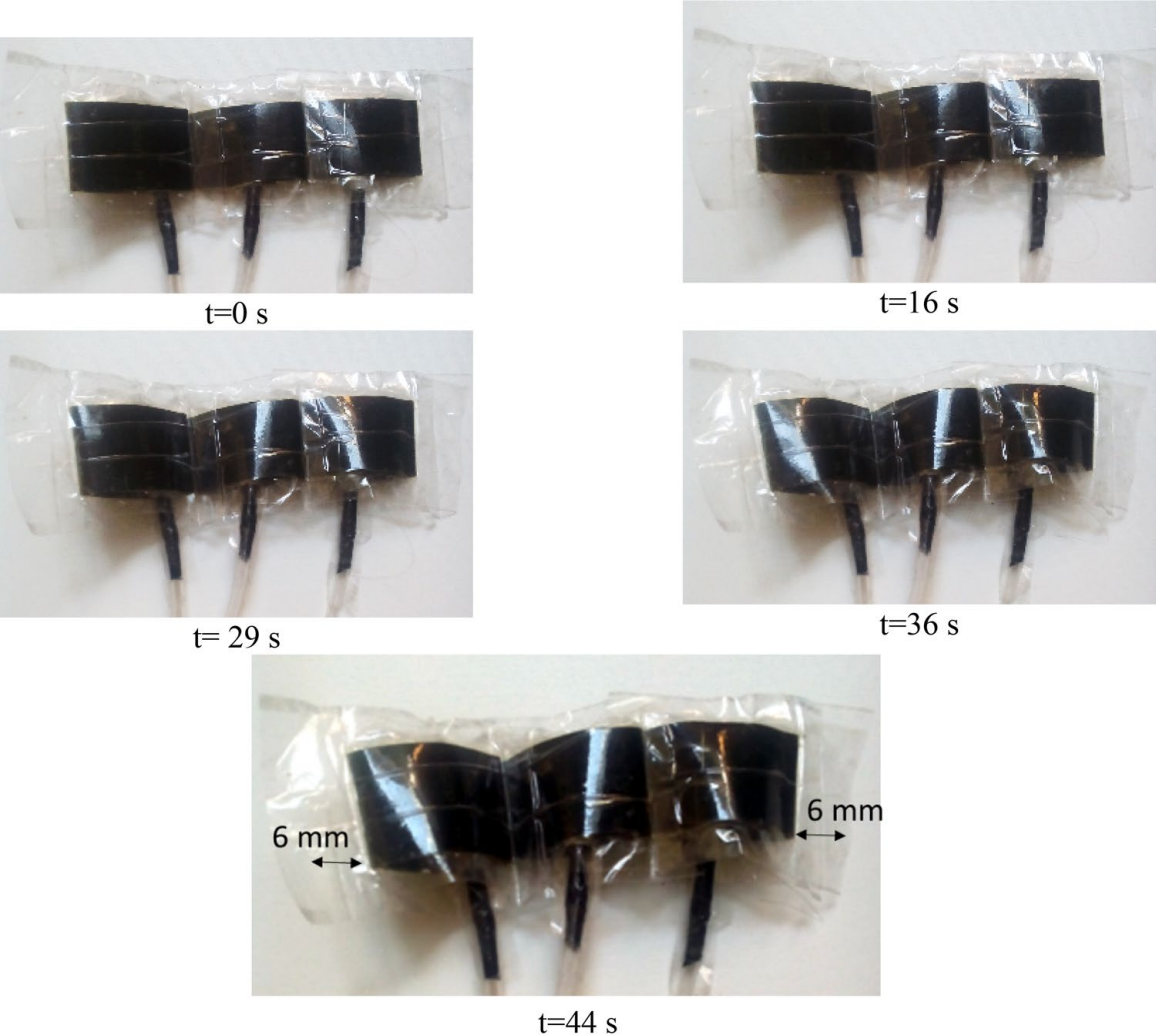
Swelling of artificial multiple soft actuator over time for the inactive mode in 12 V.

**Figure 9:**
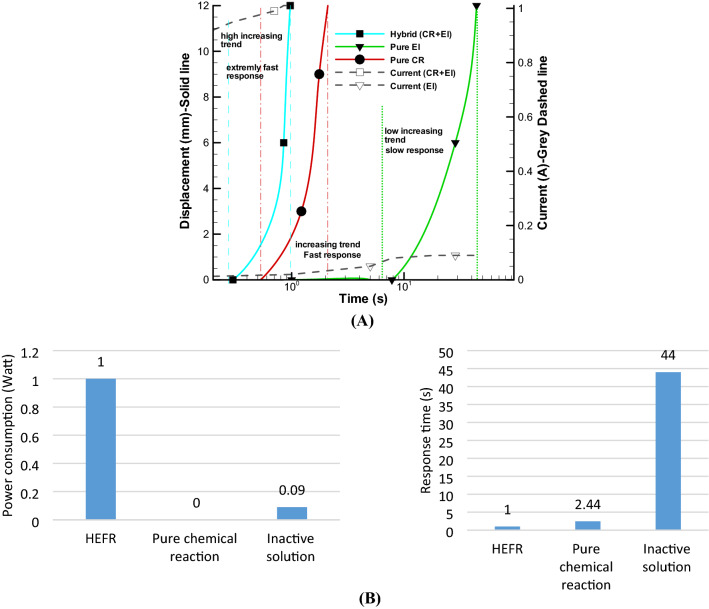
Comparison of the different displacement–time of soft actuators for chemical reaction (CR), electrolysis (EL) and Hybrid (CR + EL) modes (**A**) and bar chart of power consumption and response time of multiple actuators for different modes (**B**).

For forming larger contraction or expansion, the number of soft actuators should be increased parallel/series (greater output power/displacement) for achieving a strong artificial muscle. Based on the Fig. 8, the contraction displacement has been increased to 12 mm by connection of three soft actuators or a taped muscle which each of them has an expansion of 20 mm, and more expansion can obviously be obtained considering different applications. In the Fig. 8, swelling of a multiple soft actuator, is shown for an inactive hybrid model (12 V). To increase uniformity in swelling of three soft actuators, three separate thin pipes, which are connected parallel to one pipe, is used to enhance uniformity of pressure distribution in soft actuators. As it can be seen in Fig. 8, contraction of inactive solution has taken about a 44 s for pure electrolysis, this time will be reduced if the active mode is applied. The results of this study are compared in the state of inactive hybrid, active hybrid (HEFR) and pure chemical reaction models in the Fig. 9. The active mode provides rapid contraction/expansion approximately 2.44 times quicker than pure chemical reaction. The HEFR active hybrid method gives fast nonlinear trend at the start of reaction and amount of current consumption will raise around 6 times compared to pure electrolysis. It can be concluded that the active model is very suitable for immediate and powerful contraction (or expansion) for a quick start. Also, the passive model lack of high response time but mentioned method does not need new chemical composition. Using different modes depend on desired applications.

**Figure 10:**
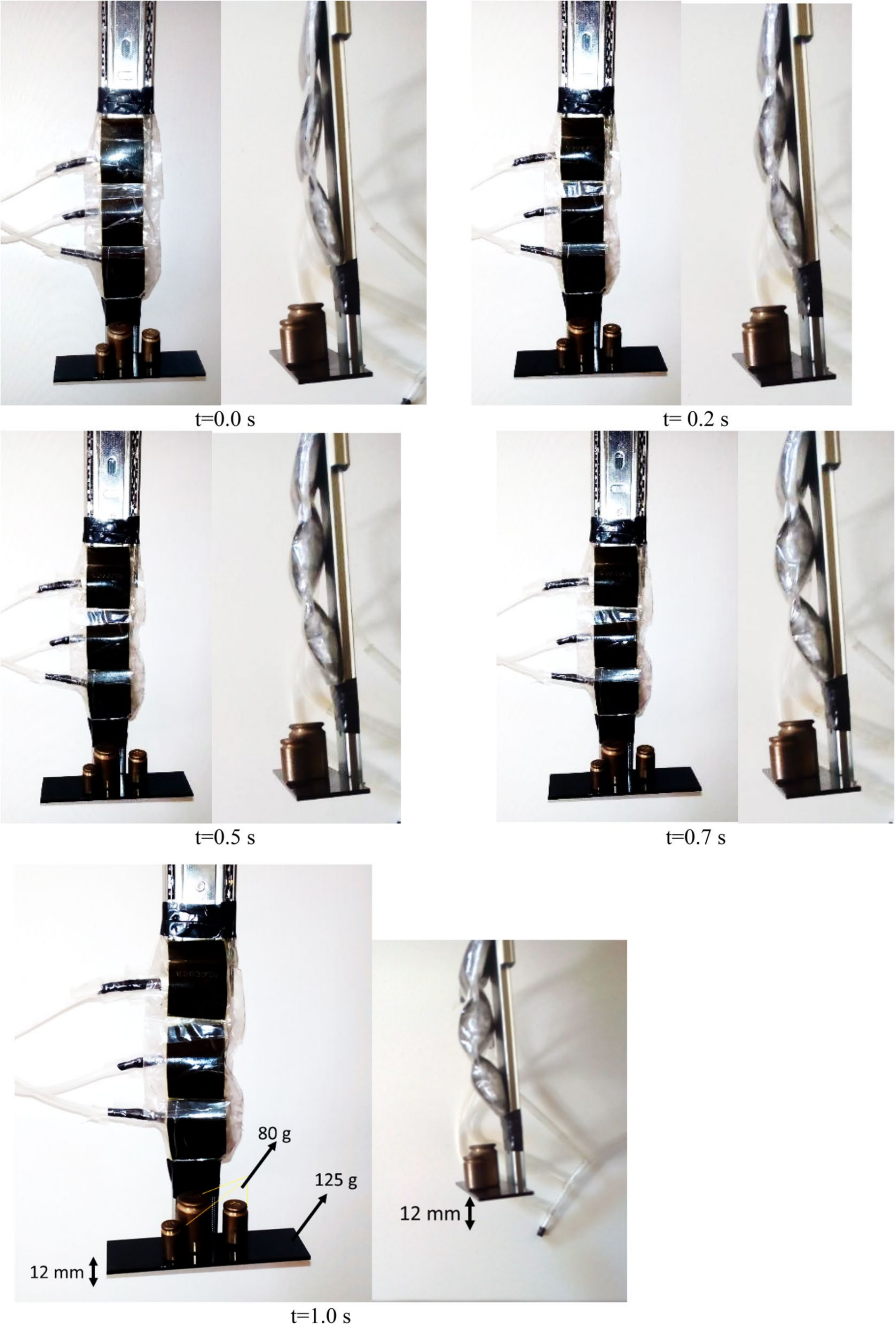
Swelling of artificial multiple soft actuators over time considering loading condition (205 g).

**Figure 11:**
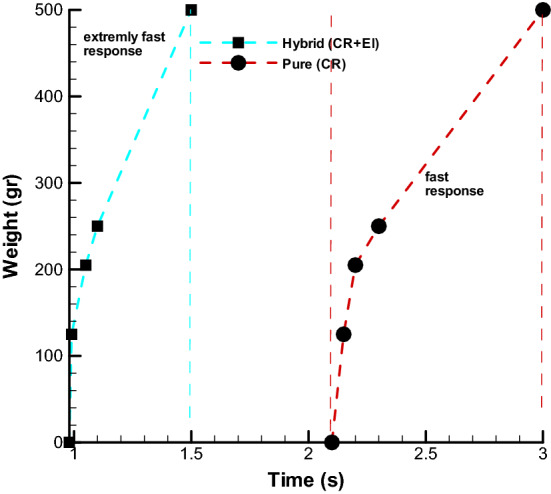
Comparison of the different response time of proposed hybrid of chemical reaction and electrolysis artificial muscle (CR + EL) and pure chemical reaction (CR) under different loading condition.

To demonstrate ability of HEFR artificial muscle, soft actuator tester mechanism in Fig. 10 is used for measuring response time of soft actuator under loading condition of 205 g (total weight). Soft actuator or myosin tester mechanism consists of sliding mechanism and net weight of sliding section is 125 g. By contraction of multiple soft actuators, the slide mechanism is displaced which is measured by the optical counter sensor (increasing encoder). For this test, weights of 125 g (weight of slide section), 205, 250 and 500 g were used and results of displacement over time are depicted in Fig. 11. Two test cases are considered including active hybrid method and pure chemical method. The response time of HEFR would be more than two times to pure chemical reaction which this fast reaction is obtained by consuming almost 12 W electricity. It is noteworthy that the total weight of the system is 33 g and the ratio of output power to weight is 7.5 times. By increasing loading condition, the nonlinear trend disappear and slow and linear trend will be govern on displacement.

### Movable wings by multiple expansion of soft actuators

Based on the previous section, we can develop this achievement in various and general applications, which in this section for example, we used HEFR method in a practical example for moving of a pair of wings. This mechanism uses a flexible diaphragm and the lifting operation is performed by a set of a taped artificial muscle that it consists of three separate soft actuators. They are folded on top of each other to increase the expansion operation. Such a mentioned mechanism is used to provide the opposite displacement direction which it situates below the diaphragm. In nature also such as proposed mechanism, one muscle is responsible for moving to one side, and another muscle is used to return to original position.

**Figure 12:**
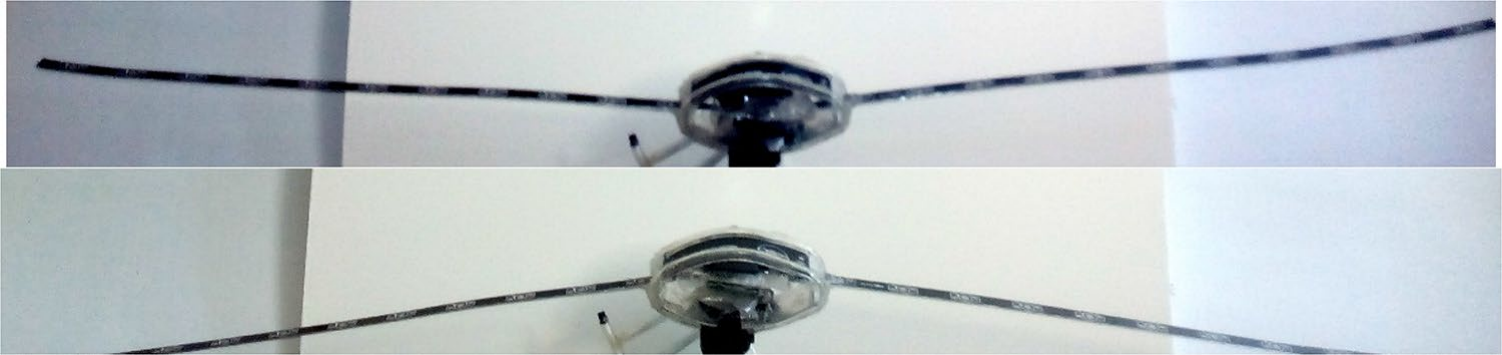
Moveable wings (upward and downward) using HEFR artificial muscles technique.

**Figure 13:**
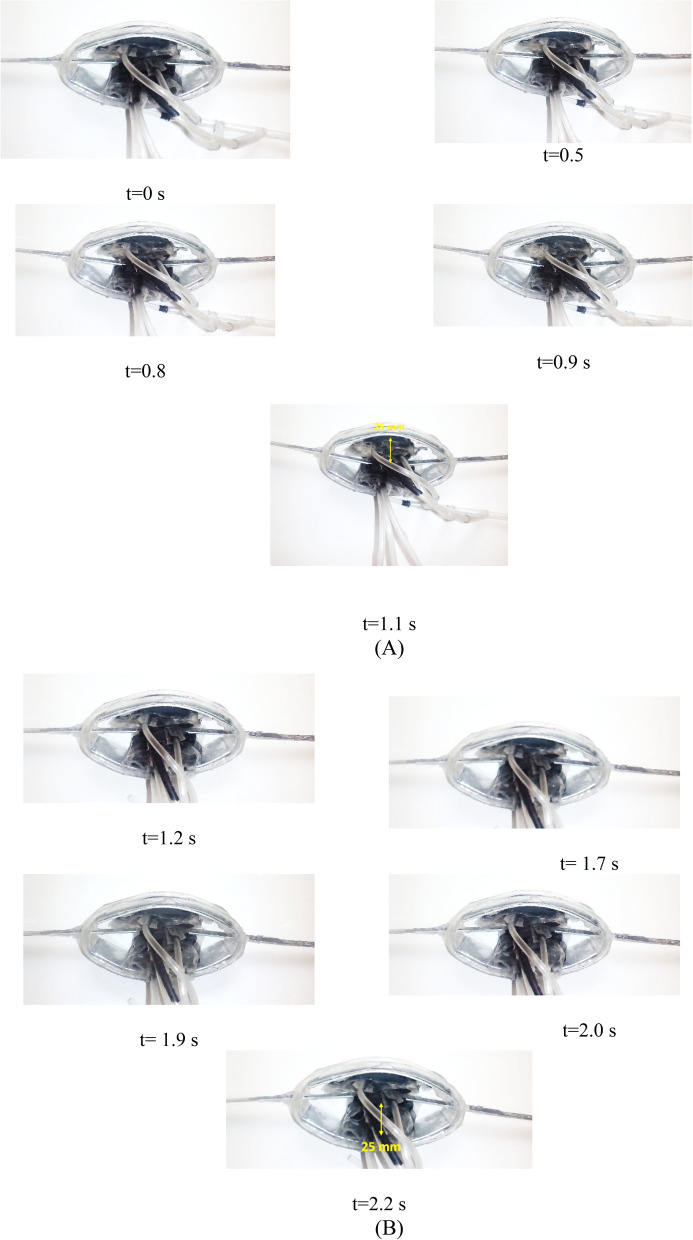
Displacement of the diaphragm over time using two sets of soft actuators up **(A)** and down **(B)** directions in HEFR mode.

Figure 12 shows the wingspan in both the up and down motion, and Fig. 13 shows the details of the artificial muscle movement. In this test, the active mode (electrolysis and chemical reaction simultaneously) is used, and in spite of all the friction between the hinges and the required torque of the wing in the model, less than 2 s will take for 25 mm displacement in upward direction. Also, this process will be repeated in the vice versa direction (Fig. 12). The power consumption for mentioned reciprocating action is almost 12.6 W. Figure 13 shows the trend of motion process (direction up and down) over time for the active and the pure chemical reaction model. As can be seen, the response time of HEFR technique almost 2.2 times quicker than pure chemical reaction. If it would be necessary, the inactive model can also be used for slower movement.

**Figure 14:**
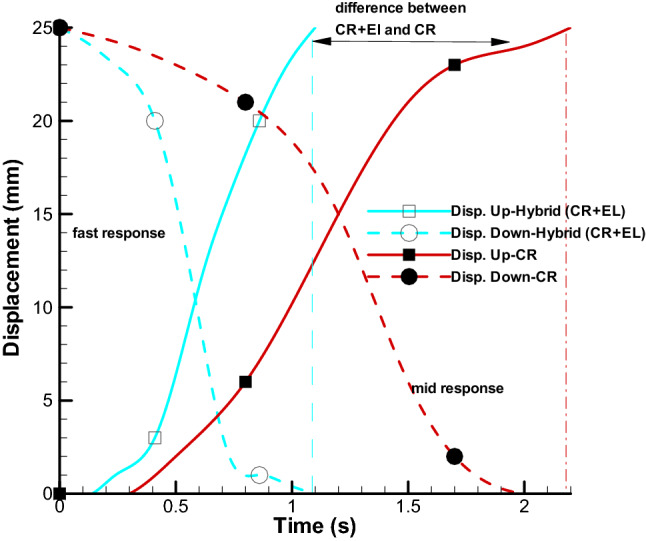
Reciprocating displacement of wings over time using two sets of soft actuators (up and down directions) in HEFR (hybrid of mixed electrolysis and fluid chemical reaction (CR + EL)) and chemical reaction mode (CR).

**Figure 15:**
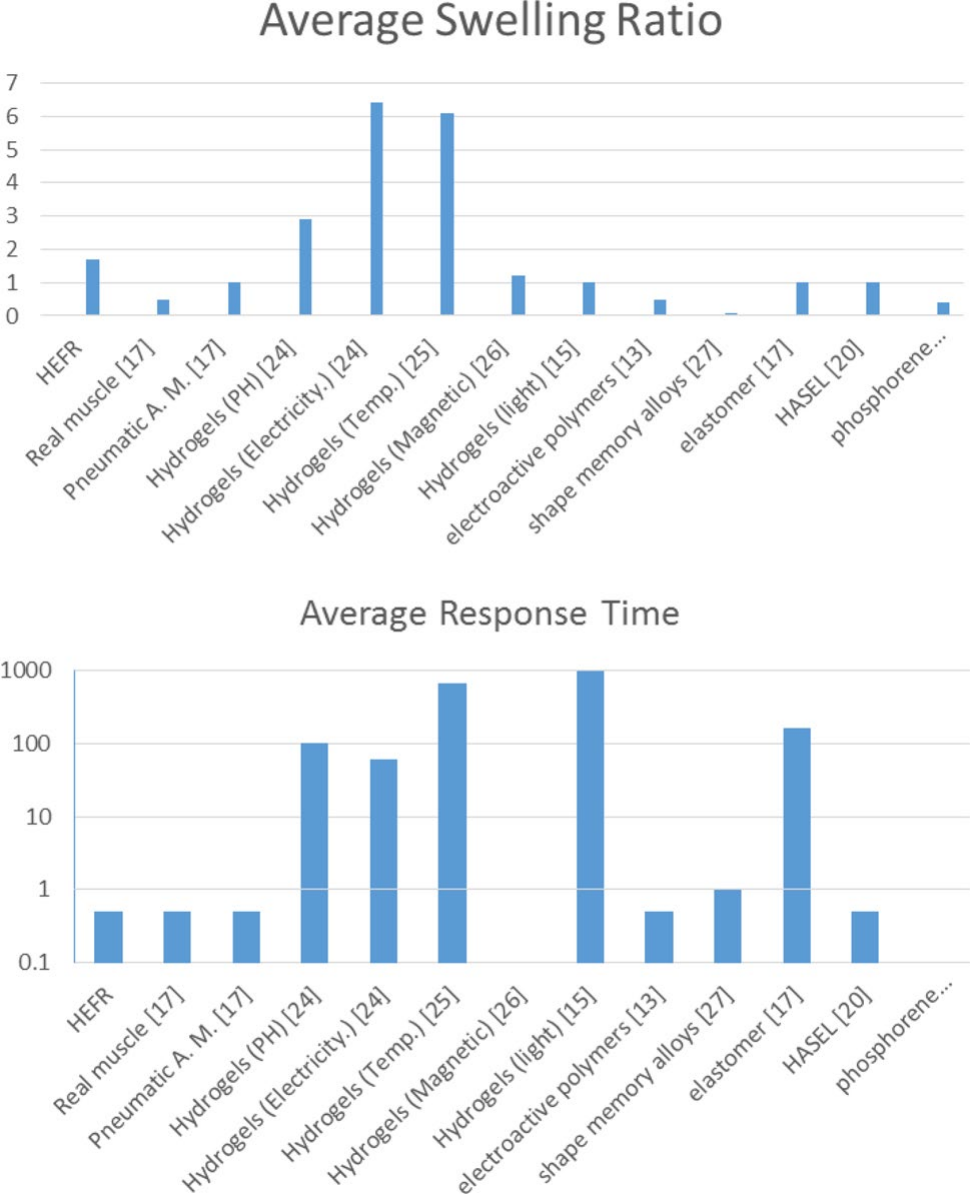
Comparison of HEFR with other methods from average swelling ration aspect **(A)** and average response time **(B)**.

By examining the present results, it can be concluded that the HEFR method has the ability to contract or expand in the proper response time, despite the resistant forces. In general, HEFR uses a combination of chemicals and electrical stimulation similar to real muscle. For comparing the present results with other methods, two criteria are considered including swelling ratio and response time in Figs. 14 and 15. By examining the present diagram, the results and two important criteria of the present method have been placed in a suitable position in comparison with the other methods and the superiority of the present method in Table 2 has been presented. It can be concluded that the HEFR method can be used in various applications.

**Table 2:** Comparison of adv. and dis adv. of HEFR with different methods.

Type	Adv	Dis adv	Ref
Present method	Close to nature, high swelling ratio, quick response time, high output force to weight ratio, cheap fabrication	Requiring chemical materials to run	–
Pneumatic A. M	High output power and high swelling ratio	Requiring high power compressor, noise pollution	^26^
Hydrogels	Can be used in different modes	Long response time	^32,33,34^
Electroactive polymers	Low volume and weight	Requiring high voltage	^17^
Shape memory alloys	Can be used without electricity	Requiring heating and cooling	^35^
Elastomer	Single self-contained mechanism	Long response time	^21^
HASEL	Acting like human muscle Single self-contained mechanism	Requiring high voltage	^24^
Materials based actuators	High performance actuators like human muscle	Proper for small scale design and more investigations are necessary	^27–29^

## Materials and methods

In this study, in order to fabric an HEFR artificial muscle that contraction or expansion is resulted from the combined electrolysis of fluids as well as their chemical reaction, the fabrication operation was divided into three general parts:

- Making soft actuator: two main criteria are considered in the manufacturing including lack of leakage in high pressure and designing the mechanism close to natural muscle. For fulfilling the first criteria, the three main materials for manufacturing are applied which a thin layer of nylon plastic (made of nylon polyEthylene (PE) materials with thickness of 85 microns (0.085 mm)) is considered for avoiding any leakage, also, a thin protective layer of ordinary adhesive tape is used for preventing any rupturing of the mentioned thin layer and a thick protective layer of thin rubber (polyvinylidene fluoride with thickness of 1 mm) is employed for forbidding any tearing of thin layers. Also, based on the myosin motion in natural muscle, in this research, the thin protective layer is folded from the middle to turn the wave motion into an inflating motion and it provides a displacement which the momentum will be transferred to solid structure.
  The method of construction is that the thin layer or nylon plastic (PE) is cut and pressed by the heating element in the form of a sealed small bag (15 × 50 mm), then it is protected by the next thin layer. The present strip should be folded from the middle. Then the tape is inserted into thick cover or thin rubber (polyvinylidene fluoride). Thick cover is cut as a cylindrical form in dimensions of 40 (length) × 30 (diameter) mm. It should be noted that the ends of the thin layers is connected to the fluid transfer line and it should be glued very well with the waterproof adhesive. For attaching more soft actuators, it is enough to be connected to the cells by pinstripe tape.
- Fabrication of force module: it includes an electrolysis module and inlet port for injection of chemical materials. The electrodes are a simple double iron cylindrical blade with radius of 1.5 mm and height of 20 mm that they are inexpensive and easy to replace in the case of corrosion. Maximum capacity for electrolysis tank is 20 mL. All of the fluid lines are rubber lines with diameter of 5 mm. The chemicals materials are sodium bicarbonate (NaHCO3 (s)) and acetic acid (CH3COOH (l)) as the reactive fluids that enough pressure is generated for contraction and expansion operations. The maximum rate of acetic acid injection on sodium bicarbonate is 1 mL/s.
  The construction method is such that the chamber is prepared in the size of 20 ml and two metal blades with a distance of 1 mm are carefully installed. Note that the wiring and connection of the blade must be done with the utmost care and heat-resistant adhesive must be used, because the temperature in the chamber has risen somewhat and if there is at the least amount of leakage, the whole compression process will be released. There must also be a chamber on one side to the fluid inlet, and one side should be the outlet. The pressure relief system also includes a servo motor model SG-90 which it moves by taking command and the pressure chamber will be released. The diameter of all lines is 5 mm.
- Building the laboratory models: a soft actuator tester mechanism is built for investigation of soft actuator behavior on the different loading conditions. The soft actuator tester consists of a sliding testing mechanism which the slide will be moved up by swelling of soft actuator and the optic sensor will count the bright spots (linear encoder: opto counter, LM393 and AVR-8mega microchip), using 1 mm shift between each bright spot on the dark strip, optic sensor can calculate the displacement. Also, the moving wing mechanism consists of two flexible aluminum wings with span 940 mm that are connected to each other in the center by a flexible diaphragm. The distance between each hinge to center of diaphragm is 55 mm. This material also uses for attaching the two wings at center. The material for attaching the wing to hinge, which should have enough flexibility for flapping action, is Otto Seal Aquarium Silicone S28. At the top and bottom of the diaphragm, there are several folded multiple soft actuator and a thin protective adhesive layer is used for the jointing the soft actuator which the construction process was explained.

## Conclusion

In this study, method of HEFR (Hybrid of Mixed Electrolysis and Fluids Chemical Reaction) artificial muscle was presented, which this method used the combination of released energy from chemical reaction of sodium bicarbonate and acetic acid as well as the released energy caused by the electrolysis of the mentioned electrolyte solution, thus the energy, as gas pressure, entered the soft actuator. Soft actuator to be able applicable for general purpose, was presented in three forms: single cell, multiple contraction cells, and several expansion cells. The main achievement are listed as follows:

- The response time of single soft actuator for HEFR mode was so quick (less than 1 s) and power consumption, considering electrolysis module in this experiment, was almost 12 Watt. This method had ability to provide 25 mm displacement for flapping wings test case in 2.2 s.
- The contraction model of several soft actuators in HEFR mode was tested under different load conditions, and for the geometry and conditions which were used in this study, the weight of less than 33 gr for a soft taped actuators in the active mode provided a suitable response time of less than 2 s.
- The pure chemical reaction had the second place from short response time aspect (2.2 times slower than HEFR)
- Also, it had been shown that although the hybrid of electrolysis with active fluid chemical reaction had a very short response time, but it required a fresh chemical reaction, while the inactive chemical reaction mode of electrolysis had a longer response time and it did not require a new chemical reaction.

It can be concluded that the HEFR artificial muscle has the ability to contract or expand with the appropriate response time and it can have desired output force for various applications of soft robotics.

## Data Availability

The authors declare that the data supporting the findings of this study are available within the paper and additional data on methods used are available upon reasonable request.

## References

[1] Hall JE (2015). Guyton and Hall Textbook of Medical Physiology (Guyton Physiology).

[2] Singth H, Singh L, Yadav M (2018). Fundamentals of Medical Physiology.

[3] Zakeri R (2021). Towards bio-inspired artificial muscle: A mechanism based on electro-osmotic flow simulated using dissipative particle dynamics. Sci. Rep..

[4] Jafari S, Zakeri R, Darbandi M (2018). DPD simulation of non-Newtonian electroosmotic fluid flow in nanochannel. Mol. Simul..

[5] Zakeri R (2019). Dissipative particle dynamics simulation of the soft micro actuator using polymer chain displacement in electro-osmotic flow. Mol. Simul..

[6] Zakeri R, Lee ES (2021). Simulation of nano elastic polymer chain displacement under pressure gradient/electroosmotic flow with the target of less dispersion of transition. Sci. Rep..

[7] Baker JS, McCormick M, Robergs RA (2010). Interaction among skeletal muscle metabolic energy systems during intense exercise. Nutr. Metab..

[8] Mitsui T, Ohshima H (2012). Theory of muscle contraction mechanism with cooperative interaction among cross bridges. Biophysics.

[9] Krans, JL. The sliding filament theory of muscle contraction. *Nat. Educ.* **3**(9), 66. https://www.nature.com/scitable/topicpage/the-sliding-filament-theory-of-muscle-contraction-14567666/ (2010).

[10] Mitsui T, Ohshima H (2021). Modeling muscle contraction mechanism in accordance with sliding-filament theory. Encycl. Biocolloid Biointerface Sci..

[11] Tolley MT (2014). A resilient, untethered soft robot. Soft Robot.

[12] Bai K, Luo M, Li T (2018). The impulse excitation joint servo drive design and adaptive backstepping control of humanoid robots. J. Bionic Eng..

[13] Swamardika A, Budiastra IN, Setiawan N, IndraEr N (2017). Design of mobile robot with robotic arm utilising microcontroller and wireless communication. Int. J. Eng. Technol..

[14] Chunbao L, Luquan Y, Ren R (2019). A review of biological fluid power systems and their potential bionic applications. J. Bionic Eng..

[15] Wang L, Qu X, Meng Q, Yuan P, Wang M (2002). Motor driving leg design for bionic crab-like robot. J. Mar. Sci. Appl..

[16] Abdellatif A, Alfayad S, Hildebrandt AC, Ouezdou FB, Mechbal N, Zweiri Y (2018). Development of a new hydraulic ankle for HYDROïD humanoid robot. J. Intell. Rob. Syst..

[17] Yang T (2018). A soft artificial muscle driven robot with reinforcement learning. Sci. Rep..

[18] Yeom SW, Oh K (2009). Biomimetic jellyfish robot based on ionic polymer metal composite actuators. Smart Mater. Struct..

[19] Lancia F, Ryabchun A, Nguindjel AD, Kwangmettatam S, Katsonis N (2019). Mechanical adaptability of artificial muscles from nanoscale molecular action. Nat. Commun..

[20] Schaffner M (2018). 3D printing of robotic soft actuators with programmable bioinspired architectures. Nat. Commun..

[21] Miriyev A, Stack K, Lipson H (2017). Soft material for soft actuators. Nat. Commun..

[22] Acome E, Mitchel SK, Morrissey TG, Emmett MB, Benjamin C, King M, Radakovitz M, Keplinger C (2018). Hydraulically amplified self-healing electrostatic actuators with muscle-like performance. Science.

[23] Kellaris N, Venkata V, Smith G, Mitchell S, Kiplinger C (2018). Peano-HASEL actuators: Muscle-mimetic, electrohydraulic transducers that linearly contract on activation. Sci. Robot.

[24] Kellaris N, Venkata V, Rothemund P, Keplinger C (2019). A Department of Mechanical Engineering, University An analytical model for the design of Peano-HASEL actuators with drastically improved performance. Extreme Mech. Lett..

[25] Martinez GJ, Tyagi M, Alijanianzadeh M, Turner A, Jager E (2019). Artificial muscles powered by glucose. Adv. Mater..

[26] Li S, Vogt DM, Rus DW, Wood RJ (2017). Fluid-driven origami-inspired artificial muscles. PNAS.

[27] Wu B, Deng HX, Jia X, Shui L, Gao E (2020). High-performance phosphorene electromechanical actuators. npj Comput. Mater..

[28] Wu B, Cai X, Shui L, Gao E, Ze LZ (2021). Extraordinary electromechanical actuation of Ti2C MXene. J. Phys. Chem..

[29] Rogers G, Liu J (2012). High-performance graphene oxide electromechanical actuators. Am. Chem. Soc..

[30] Zakeri R, Zakeri R (2021). Deformable airfoil using hybrid of mixed integration electrolysis and fluids chemical reaction (HEFR) artificial muscle technique. Sci. Rep..

[31] Carlson GL (1990). A new approach to the baking soda-vinegar reaction. Chem. Educ..

[32] Shim TS, Kim SH, Heo CJ, Jeon HC, Yang SM (2012). Controlled origami folding of hydrogel bilayers with sustained reversibility for robust microcarriers. Angew. Chem..

[33] Yoshida R (1995). Comb-type grafted hydrogels with rapid deswelling response to temperature changes. Nature.

[34] Kondaveeti S, Semeano ATS, Cornejo DR, Ulrich H, Petri DF (2018). S, Magnetic hydrogels for levodopa release and cell stimulation triggered by external magnetic field. Colloids Surf. B Biointerfaces.

[35] Potapov PL, Da Silva EP (2000). Time response of shape memory alloy actuators. J. Intell. Mater. Syst. Struct..

